# Hydrogen Storage for Mobility: A Review

**DOI:** 10.3390/ma12121973

**Published:** 2019-06-19

**Authors:** Etienne Rivard, Michel Trudeau, Karim Zaghib

**Affiliations:** Centre of Excellence in Transportation Electrification and Energy Storage, Hydro-Quebec, 1806, boul. Lionel-Boulet, Varennes J3X 1S1, Canada; trudeau.michel@hydro.qc.ca

**Keywords:** hydrogen mobility, hydrogen storage, storage systems assessment, Kubas-type hydrogen storage, hydrogen economy

## Abstract

Numerous reviews on hydrogen storage have previously been published. However, most of these reviews deal either exclusively with storage materials or the global hydrogen economy. This paper presents a review of hydrogen storage systems that are relevant for mobility applications. The ideal storage medium should allow high volumetric and gravimetric energy densities, quick uptake and release of fuel, operation at room temperatures and atmospheric pressure, safe use, and balanced cost-effectiveness. All current hydrogen storage technologies have significant drawbacks, including complex thermal management systems, boil-off, poor efficiency, expensive catalysts, stability issues, slow response rates, high operating pressures, low energy densities, and risks of violent and uncontrolled spontaneous reactions. While not perfect, the current leading industry standard of compressed hydrogen offers a functional solution and demonstrates a storage option for mobility compared to other technologies.

## 1. Introduction

According to the Intergovernmental Panel on Climate Change (IPCC), it is almost certain that the unusually fast global warming is a direct result of human activity [1]. The resulting climate change is linked to significant environmental impacts that are connected to the disappearance of animal species [2,3], decreased agricultural yield [4,5,6], increasingly frequent extreme weather events [7,8], human migration [9,10,11], and conflicts [12,13,14]. 

To mitigate the progression of climate change, there is an increasing momentum to reduce the global emissions of greenhouse gases. For example, France approved the law no. 2015-992, which requires a 40% reduction of greenhouse gases by 2030 compared to 1990 [15]. Although the use of fossil fuels is not the only source of greenhouse gases, it is certainly a major one. According to the United States Environmental Protection Agency, fossil fuels account for 76% of all U.S. emissions due to human activities [16]. It can be assumed that a significant reduction of greenhouse gas emissions implies the reduction of fossil fuel usage. However, that is not a simple task because products derived from fossil fuels are not just energy carriers, they are also a primary source of energy. Hydrogen is an energy carrier that should be produced by an environmentally clean process in order to have a truly positive impact on decarbonization. In 2017, more than 85% of the energy produced globally came from fossil fuels [17]. Therefore, if the world were to completely switch over to a hydrogen economy that eliminates all fossil fuel consumption, an energy shortage would soon occur [18,19,20,21,22]. This aspect represents a significant problem to find suitable energy sources. However, this topic will not be discussed in this study. Instead, this study focuses on the use of hydrogen as a renewable energy storage medium [23,24,25,26,27]. 

Renewable energy is theoretically plentiful. Nakićenović et al. [28] estimated the minimum annual solar energy potential to be 1575 EJ, which would exceed the annual global energy consumption by approximately 566 EJ [17]. However, there are other issues related to these energy alternatives, such as high system costs [29]. For instance, price reductions on components such as solar panels and wind generators no longer have a significant impact on the price of energy because their cost is small when compared to installation and other system-related costs. Typically, there is also a mismatch between production capacity and demand, resulting in overproduction or shortage. Storing energy in the form of hydrogen can coordinate production and consumption [30,31,32]. Each hydrogen storage technique possesses its own characteristics, such as energy density, speed of kinetics, and efficiency. Therefore, it is difficult to identify a single solution to all storage needs. 

Numerous reviews on hydrogen storage have been published [33,34,35,36,37,38]. However, most of these reviews deal either exclusively with storage materials or the global hydrogen economy [39,40,41,42,43]. There are varied requirements for hydrogen storage, depending on the application. Therefore, in this paper, we evaluate the hydrogen storage options for mobility applications. For transportation, refueling must be fast, safety is of prime importance, and the weight and size of the storage system should be as low as possible. It is important to consider the whole system to produce a practical solution that the industry can accept. Moreover, cost and efficiency of batteries and hydrogen systems are compared for a deeper analysis.

## 2. Hydrogen for Mobility

Before analyzing hydrogen storage methods for mobility applications, it is essential to discuss if there is a real need for such solution, considering present battery technologies.

### 2.1. Overall Efficiency

Losses occur at each energy conversion step. For hydrogen, those steps mainly include production, storage, and utilization. The current efficiency of water electrolysis can reach 86% with heat recovery [44]. The energy required to compress hydrogen to 700 bar and deliver it to a vehicle can vary between 5% and 20% of the hydrogen lower heating value [45]. Proton-exchange membrane (PEM) fuel cells can achieve an efficiency of approximately 60% [46]. This yields a combined efficiency that may vary between 41% and 49%. According to the United States Department of Energy (DOE), electric vehicles are approximately 59–62% efficient in the conversion of energy from the electric network to the mechanical work at their wheels [47]. Therefore, the efficiency of battery vehicles can be increased, but the room for improvement is small.

### 2.2. Costs of Battery vs. Fuel Cell

Although batteries and fuel cell systems are complementary in many applications, they are often seen as competing technologies. Therefore, a comparison is unavoidable, especially from an economic perspective. Prices vary according to battery chemistry, but 270 $/kWh [48] is a fair price estimate for a lithium-ion battery. Assuming that a battery charges and discharges at a rate of 1 C, which is equivalent to 1 hour, the specific battery cost would be 270 $/kW in terms of power output. Compressed hydrogen tanks and fuel cell stacks currently cost approximately 15 $/kWh (see Section 3) and 100 $/kW [49], respectively. Therefore, hydrogen vehicles are the most affordable of these two options. Regarding refueling, it is possible that hydrogen prices at the pump are reduced to 8 $/kg [50]. That would be equivalent to 0.24 $/kWh which is cheaper than the price of electricity in many developed countries. 

### 2.3. Practical Advantages of Fuel Cells

Hydrogen vehicles can be refueled in less than 10 min, which is a considerable advantage, especially for high use factor applications. Weight is also significantly lower at approximately 550 Wh/kg versus 150 Wh/kg for batteries [51]. These advantages are some of the main reasons that hydrogen and fuel cells are interesting for heavy vehicles, such as buses and trucks.

Many battery types contain metals such as cobalt, whose extraction can involve human health hazards and negative impacts to the environment. Materials needed for PEM fuel cells, including polymers and graphite, are common and safe, except for platinum. Platinum is an excellent low-temperature catalyst, which is very difficult to replace. A shortage of Pt would be feared upon widespread adoption of PEM fuel cells, but according to Heraeus, the demand projected for 2020 can be easily met [52]. 

## 3. Ideal Storage Method

Before the evaluation of hydrogen storage techniques, an ideal storage medium for mobility can be defined by qualifying or quantifying the characteristics of each system. High volumetric and gravimetric energy densities are clearly desirable for mobile applications. Gasoline and diesel are currently the ubiquitous fuels for surface transportation, and they can be used as a benchmark. The energy densities of these fuels vary because complex mixtures and different blends are available on the market. However, values close to 38 wt % and 35 MJ/L are typical. Pure hydrogen at ambient temperature and pressure offers excellent gravimetric but poor volumetric energy densities of 120 MJ/kg (100 wt %) and 0.01 MJ/L, respectively. Some physico-chemical properties of hydrogen and natural gas are compared in Table 1. Another important performance metric is the speed of kinetics. This term designates the rate at which the system can release hydrogen upon demand and stop this release when required. The rates should match transportation applications, e.g., acceleration and braking of an automobile. In addition to that, for mobility, a high power output battery is needed for peak demands. 

Temperature-dependent hydrogen storage techniques imply the addition of a heat management system [56,57,58,59,60], which adds costs, complexity, and possibly mass. Ideally, this technique should be avoided, and operation near ambient temperature throughout refueling, standby, and discharge is desirable. Another important aspect to consider is the operating pressure. Pressure vessels must be reinforced with high strength materials that are subject to strict regulation and testing, which negatively impacts gravimetric density and costs. The final important thermodynamic property is efficiency. If hydrogen is used in an effort to capture renewable energy and displace hydrocarbons, then efficiency should be as high as possible to make optimal use of available renewable energy.

For an ideal storage method, safety is essential, especially for general public use. Toxicity, flammability, danger of explosion or projections, etc., are not desirable, but they are difficult to quantify. The use of materials that require resource intensive extraction or designs that make recycling difficult or impossible should also be avoided.

All the aforementioned characteristics should be affordable to ensure market penetration.

DOE targets on hydrogen storage [61] provide a good prospect of the expected potential performance. These targets are summarized in Table 2.

## 4. Present Industry Choice: Compressed Gas

**Figure 1:**
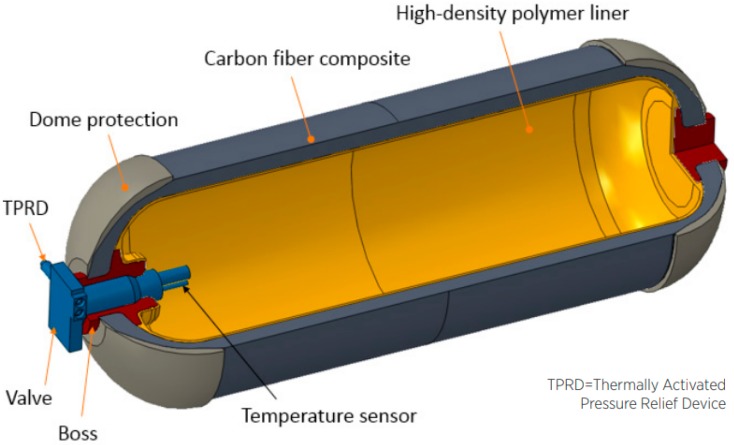
Type-IV composite overwrapped hydrogen pressure vessel (source: Process Modeling Group, Nuclear Engineering Division. Argonne National Lab (ANL)). Reprinted from Ref. [67]; Copyright DOE 2017.

Compressed gas is the most well-established hydrogen storage technology. As evidence of that, the Society of Automotive Engineers established the standard SAE J2600 on Compressed Hydrogen Surface Vehicle Fueling Connection Devices [62], which should be applied to the “design and testing of Compressed Hydrogen Surface Vehicle (CHSV) fueling connectors, nozzles, and receptacles.” Commercial fuel cell electric vehicles such as the Toyota Mirai and the Honda Clarity both rely on pressure vessels for onboard hydrogen storage [63]. Pressure vessels are classified according to types. Table 3 provides a summary of the features for each type [64], and Figure 1 provides a schematic view of a type IV pressurized hydrogen reservoir.

According to Züttel [33], the gravimetric density of high-pressure gas cylinders is 13% at pressure of 800 bar. In contrast, according to the Toyota Motor Corporation, the gravimetric density of the 2017 Mirai tank is 5.7 wt % [68] at 700 bar. The Mirai tank has an internal volume of 122.4 L, with volumetric energy density up to 4.90 MJ/L. This significant difference shows that even for established commercial technologies, performance may vary widely depending on the application. Energy is required to compress hydrogen, and it takes a minimum of 4.1 wt % to compress hydrogen from 20 to 700 bar [45]. All gases, hydrogen included, release heat when compressed. A common strategy to avoid overheating the tank during refill by compression is to cool the gas beforehand [69]. This requires an additional 1.8–3.6 wt % [45] for hydrogen pre-cooling. However, there is no need for a thermal management system onboard the vehicle.

The pressure is extremely high and demands an extremely robust tank. This limits the shape of the tank to a cylinder and makes its integration into the vehicle architecture more difficult. The kinetics of compressed gas are ideal, and the fuel flow can increase or decrease in a virtually limitless manner. 

From a safety point of view, the typical materials involved, such as carbon fiber and nylon-6 [70], are not toxic or environmentally harmful. High pressure, however, always represents a risk [71]. 

## 5. Other Storage Methods

### 5.1. Liquid Hydrogen

The basic requirement for liquid hydrogen (LH_2_) storage is to reduce its temperature to −253 °C, which is the boiling point of dihydrogen at ambient pressure [72]. A liquid hydrogen tank is typically not designed to withstand internal pressure, but rather to hold a cryogenic liquid [73]. The vessel must be properly insulated to reduce heat transfer to a minimum [74]. Heat transfer from the environment to the liquid increases the pressure inside the tank. Since the tank is not designed to hold high pressure, hydrogen is allowed to escape through a relief valve, which is sometimes referred to as “boil-off” [75,76,77]. Because thermal insulation is never perfect, an unused hydrogen reservoir stored in a warm environment will eventually deplete itself. Liquid hydrogen storage is a mature technology and is the basis of the existing industrial infrastructure network for storage and delivery. As an example, Amos [78] published a comprehensive report in 1998 on the “Costs of Storing and Transporting Hydrogen,” including extensive information on LH_2_.

Large cryogenic hydrogen tanks tend to minimize the proportion of insulation mass and volume with respect to hydrogen volume [79]. The geometrical shape that allows the largest volume to surface area ratio is the sphere. A high-volume-to-surface ratio can minimize heat transfer, which is responsible for the boil-off effect. A hypothetical spherical tank surrounded by 25 mm of insulation material, capable of holding 5 kg of hydrogen, does not exceed volumetric and gravimetric energy densities of 6.4 MJ/L and 7.5 wt % [80], respectively. The kinetics are not problematic and are comparable to those of compressed hydrogen. 

The thermodynamic aspect of liquid hydrogen makes it less attractive for mobility. At −253 °C, the low storage temperature is a problem mainly due to boil-off losses. Liquid hydrogen tanks do not have to withstand high pressure, but they must be heavily insulated, which results in reservoirs with thick walls. According to the U.S. Drive [81], the costs associated with hydrogen liquefaction reach approximately 1.00 $/kg because the plants are “capital and footprint intensive”. In 2009, the best plant in the USA achieved an efficiency of 70% [82], which is still a considerable energy penalty for storage. 

Lower and higher costs are associated with larger and smaller reservoirs, respectively, at either end of this broad spectrum. Petitpas and Simon [83] reported a specific cost of 167 $/kg for a capacity of 4300 kg, whereas Meneghelli et al. [84] estimated 386 $/kg for a 100 L internal volume reservoir for automotive applications. The costs of storage tanks are not discussed in this paper, but it is important to note that the cost of hydrogen liquefaction is significant, both in terms of energy and equipment.

Liquid hydrogen is applicable where high energy density is required and boil-off is less of a concern. Commercial aircrafts could be considered, since they have intensive service and refuel is done at designated locations. However, the volumetric energy density of liquid hydrogen is almost 4 times lower than that of kerosene [85,86], even excluding the volume required for insulation. Therefore, liquid hydrogen has an unacceptably short range for a commercial airliner.

### 5.2. Cold/Cryo Compression

**Figure 2:**
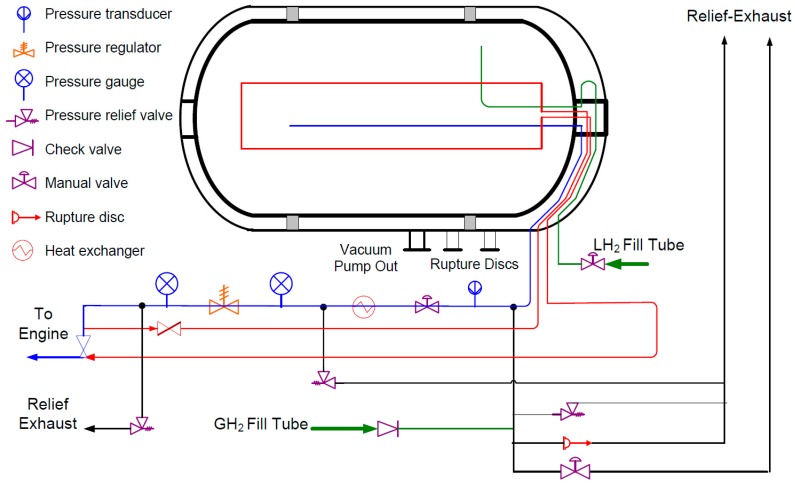
Design schematic of the Lawrence Livermore National Laboratory Gen-3 cryo-compressed H_2_ storage tank system. Reprinted from Ref. [90] with permission; Copyright Argonne National Laboratory 2009.

Cold/Cryo compression of hydrogen is considered a hybrid method that combines compressed gas and liquid hydrogen [87]. The tank must be designed to hold a cryogenic fluid and, therefore, to withstand internal pressure. The diagram shown in Figure 2 provides an example of such a device. According to Petitpas and Simon [83], cryo-compressed H_2_ (CcH_2_) storage systems have high density and feasible costs, that "scale well with capacity". 

Kircher and Kunze [88] from BMW described a prototype capable of gravimetric and volumetric energy densities of 5.4 wt % and 4.0 MJ/L, respectively, with a hydrogen boil-off rate of 3–7 g/h. Because of the large capacity of the tank, a significant amount of hydrogen fuel would still be left in the tank, even after an extended idling period [89]. Moreover, kinetics are not an issue with this technology because there are no physicochemical bonds to break to release hydrogen.

The research by Meneghelli et al. [84] is another good example of a cryo-compressed tank for mobile applications. Their system operates at 40–80 K and 300 bar. From an efficiency standpoint, cryo-compression can be considered superior to liquid storage alone because boil-off is curbed. Ahluwalia et al. [91] reported a dormancy period of more than 7 days without loss when the reservoir was filled at 85% of its maximum capacity. From an environmental standpoint, it is difficult to foresee major issues. In fact, when compared with 700 bar room-temperature storage, cryo-compression requires less high strength materials [92]. Insulation is normally achieved with vacuum [93], and pressure is contained by means of high-strength materials, both of which are not typically rare or harmful [94]. Cost is expected to be in the range of 390 $/kgH_2_ [84]. 

### 5.3. Metal–Organic Framework

**Figure 3:**
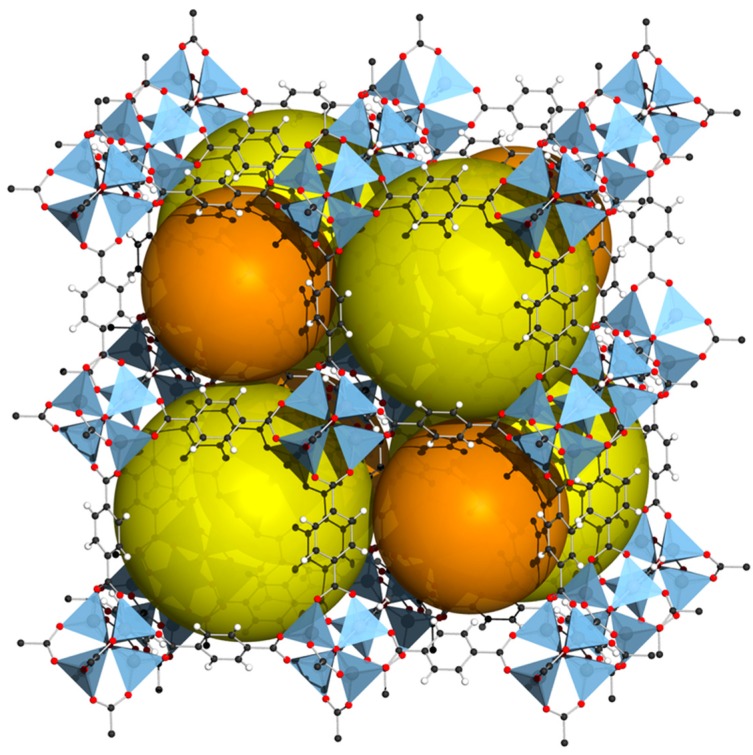
Crystal structure of MOF-5. Reprinted from Ref. [106]; Copyright Wikipedia 2018.

Metal–organic frameworks (MOFs) are a class of materials that generally work well for hydrogen storage at low temperatures of approximately 77 K. There is an enormous variety of MOFs that are engineered for different applications, including fuel storage [95], batteries [96], supercapacitors [97], photocatalysis [98], and phototherapy [99]. This review discusses a few examples. Rosi et al. [100] reported capacities of 4.5 wt % at 78 K and 1.0 wt % at room temperature and 20 bar, with MOF-5. Figure 3 provides a ball-and-stick representation of this framework. The yellow and orange spheres emphasize pores of the structure. The gravimetric energy density at room temperature is quite modest. The volumetric energy density can reach 7.2 MJ/L at 100 bar and 77 K [101]. MOFs are porous materials composed of crystals [102], and hydrogen must diffuse through those crystals to be stored [103]. The rate of adsorption depends on the diffusivity of hydrogen in the MOF, but also on the size of the crystals [104]. Nevertheless, the whole process is quite fast, in the order of seconds, and should not pose problems concerning refueling times. Cycling stability is possible but can be an issue [105]. 

When adsorption and absorption are triggered by a temperature variation, a thermal management system is required [107]. Many approaches are possible when designing such systems [108,109,110,111,112,113,114,115,116,117,118,119]. It is worth noting that the thermal conductivity of MOFs is approximately 0.3 W/(m·K) [120], which is extremely low. For instance, the thermal conductivity of copper is 400 W/(m·K). The low conductivity of MOFs represents an additional challenge for the thermal management in the design of MOF-based storage systems. Because the design of heat transfer devices is resource intensive, Hardy et al. [121] proposed a method to assess the overall characteristics of an optimal system. They concluded that an MOF-based hydrogen storage system requires a material that stores 4.5 times more hydrogen than MOF-5 in order to satisfy the 2025 DOE objectives. The chemical formula of MOF-5 is Zn_4_O(BDC)_3_, in which BDC stands for 1,4-benzenedicarboxylate. The addition of precious metal nanoparticles, such as platinum and palladium, into MOFs can increase their hydrogen storage capacity. Proch et al. [122] achieved a storage capacity of 2.5 wt % by adding platinum particles, but there was a sharp drop to 0.5 wt % after a few cycles.

It could be argued that MOFs combine the disadvantages of both techniques. The cryogenic temperatures imply low efficiency due to costly and energy consuming refrigeration techniques. In addition, appropriate tank insulation and thermal management systems are needed. Moreover, while the pressure is not excessively high, it still imposes the risks associated with all pressure vessels. 

Scaling up MOF production is also a challenge, which is the subject of current scientific research [123]. DeSantis et al. [124] predicted that the production costs at an industrial scale of 2.5 Mkg/year would fall between 13 $/kg and 36 $/kg. 

### 5.4. Carbon Nanostructures

The storage potential of single-walled carbon nanotubes have been calculated based on grand canonical Monte Carlo simulations, which predicted a storage capacity slightly below 10 wt % at 298 K and 10 MPa [125]. Ariharan et al. [126] synthesized carbon nanotubes doped with nitrogen. They reported a reversible storage capacity of 2.0 wt % at 298 K and 100 bar, representing a very modest capacity which is not close to the DOE goals. The pressure is also relatively high, and the addition of a storage vessel capable of withstanding the pressure would decrease the energy density. 

Kaskun and Kayfeci [127] reported a capacity of 0.298 wt % at 20 bar when multi-walled carbon nanotubes doped with nickel were used. Silambarasan et al. [128] investigated the effect of gamma-ray irradiation on the hydrogen storage properties of multi-walled carbon nanotubes. The results were also modest: 1.2 wt % at 100 °C. 

Masika and Mokaya [129] achieved a storage capacity of 7.3 wt % at 20 bar and 77 K using zeolite templated carbon. While the gravimetric hydrogen storage capacity is interesting, the storage temperature is extremely low. Similar results were obtained by other carbon structures such as a graphitic structure but also at extremely low temperatures [130,131]. The Chahine rule is a widely accepted concept which states that, in general, there is 1 wt % hydrogen adsorption for every 500 m^2^/g of surface area. 

### 5.5. Metal Hydrides

As their name implies, metal hydrides are compounds containing metal(s) and hydrogen. Magnesium hydride [132] is an attractive material for hydrogen storage because of its abundance and affordability [133]. With a density of 1.45 g/cm^3^, the energy densities of this raw material are 7.6 wt % and 13.22 MJ/L. 

Unfortunately, the high temperatures [134], high energy, and slow kinetics [135] involved in the reaction of simple hydrides are generally a problem for reversible storage. In its pure form, magnesium must be heated significantly, up to 260–425 °C, to be converted into hydride [136]. Although not high, a 20 bar pressure was also required in the experiments. Various methods can overcome the aforementioned disadvantages. For instance, Paskevicius et al. [137] reduced ${\mathrm{MgH}}_{2}$ to nanoparticles and suspended them in a LiCl salt matrix. They achieved a reduction of the equilibrium temperature of approximately 6 K when compared to that of pure magnesium hydride. However, the temperature reduction was modest and would not significantly impact the thermal management of a magnesium hydride-based hydrogen storage system. Kinetics—hydrogenation and dehydrogenation rates—can be improved by the addition of nanoparticles. Zaluska et al. [138] observed that the addition of palladium to magnesium significantly accelerates hydrogen storage kinetics. However, the use of noble metals has its drawbacks. Yahya and Ismail [139] report a storage capacity of 6.1 wt % in 1.3 min at 320 °C and 27 bar using an Ni composite. Sodium aluminium hydride (NaAlH_4_) also received attention until the late 2000s [140] but its capacity remains relatively low at 4.9 wt %. 

The efficiency of metal hydrides is not optimal. High temperatures during fueling and operation imply energy losses and bulky insulation. Rusman and Dahari [141] published a comprehensive review on metal hydrides applied to hydrogen storage. Complex hydrides operate over a broad range of temperatures. Since the hydrogenation and dehydrogenation reactions are endothermic and exothermic [142], it is unlikely that the heat generated or required by the chemical reactions can be kept in a closed loop or recycled. The safety of hydrides is also questionable. For instance, magnesium hydride is extremely reactive and may ignite when exposed to air or water [143]. 

In 2007, Sakintuna et al. [144] concluded that there was not a perfect material to store hydrogen that met the DOE goals for transport applications. They mentioned that, despite the positive results (improved kinetics, lower decomposition temperatures) for metal hydrides, there was still a need for further research to develop an optimum material.

### 5.6. Metal Borohydrides

Ley et al. [145] provide a thorough review of complex hydrides for hydrogen storage. The gravimetric and volumetric capacities for metal borohydrides ranged from 14.9 wt % to 18.5 wt %, and from 9.8 MJ/L to 17.6 MJ/L, respectively. The obtained values were significantly positive. Lithium borohydride (LiBH_4_), in particular, was widely investigated [146,147,148]. However, LiBH_4_ hydrogenation and dehydrogenation temperatures were high and the kinetics slow [149,150,151]. Reversibility is also an issue as intermediate compounds are formed during the numerous reaction steps [152].

A specific member of the metal borohydride family, the nanoporous hydride γ-Mg(BH_4_)_2_ contains 17.4 wt % of hydrogen at 105 bar and −143 °C, according to Ley et al. [145]. The hydrogen is adsorbed in the molecule, and sorption is reversible. There is no information, however, on the kinetics of the reaction. Because of the low temperature involved, boil-off could be a problem, and thermal management will likely be necessary to control the rate of hydrogen uptake and release. 

### 5.7. Kubas-Type Hydrogen

According to Skipper et al. [153] H–H bonds are not broken in the Kubas interaction [154,155], but rather lengthened. The name of the chemical bond refers to the author of the aforementioned work. The Kubas-type interaction is a low-strength chemical bond occurring with transition metals. It may be described as chemisorption since chemical reactions take place at the surface of the material. 

**Figure 4:**
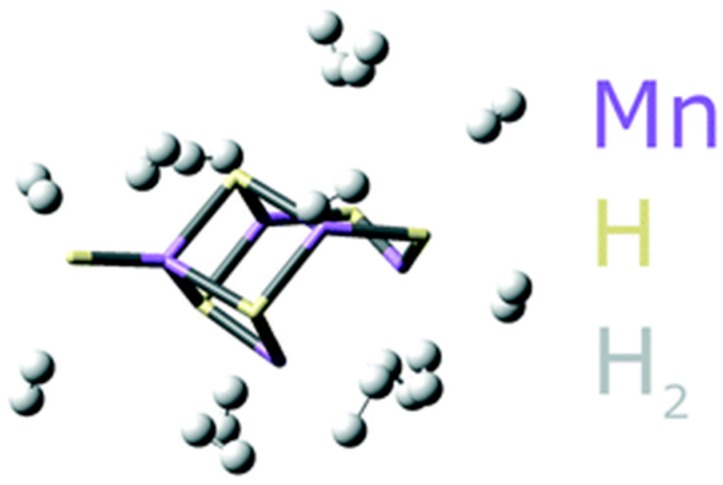
Computer simulated representation of 10 dihydrogen molecules attached to the manganese hydride basic compound in their possible binding sites. Basic compound represented as tubes while H_2_ appears as balls and sticks. Reprinted from Ref. [156]; Copyright Royal Society of Chemistry 2019.

Morris et al. [156] recently reported 10.5 wt % and 23.64 MJ/L at 120 bar and ambient temperature with a manganese hydride molecular sieve. The material showed no sign of degradation after 54 cycles. Furthermore, the reaction is thermally neutral. Adsorption is triggered by pressure variation, eliminating the need for a temperature management system. Figure 4 shows a computer simulated representation of 10 dihydrogen molecules attached to the manganese hydride basic compound in their possible binding sites. The basic compound is represented as tubes while H_2_ appears as balls and sticks. If these results are corroborated, they can lead to a system gravimetric and volumetric energy density above the DOE target. Moreover, recent density functional theory (DFT) calculations suggest that other compounds such as VH_3_ and CrH_3_ could present optimum properties [157]. Moreover, even if high-pressure is still used, it is well below the present 700 bar energy standard. It would require only a type-I or II pressure vessel, which are much cheaper than the currently used type-IV and can be designed to better fit in the vehicles. Moreover, a pressure below 200 bar would greatly simplify the infrastructure needed and, thus, reduce the costs of the hydrogen distribution network.

### 5.8. Liquid Organic Hydrogen Carriers

**Figure 5:**
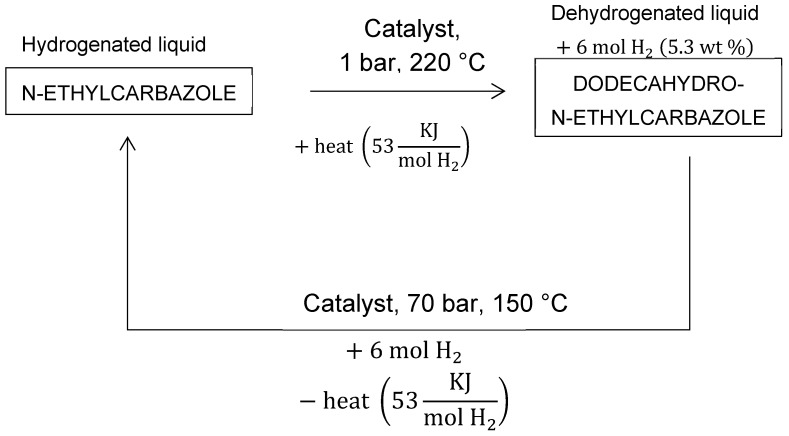
N-ethylcarbazole hydrogen uptake and release. [164].

Liquid organic hydrogen carriers (LOHC) apply the concept of hydrogenating and dehydrogenating chemical compounds to store hydrogen [158,159,160,161,162]. Figure 5 illustrates this idea with an example. The advantage of storing hydrogen in this manner is the ability to use existing infrastructure, such as tankers and tanker trucks. He et al. [163] published a comprehensive review on this topic. 

Teichmann et al. [164] published a study that focused on heterocyclic aromatic hydrocarbons LOHCs. The strategy with LOHCs is to hydrogenate an organic compound to store hydrogen, and dehydrogenate it when hydrogen is needed. Handling this type of carrier is much easier than managing compressed gas [165]. In this context, dodecahydro-N-ethylcarbazole has been widely studied [166,167,168,169,170]. When dehydrogenated, the substance becomes N-ethylcarbazole. It holds a theoretical maximum of 8.5 wt % hydrogen [171]. This corresponds to approximately 7 MJ/L, which is quite comparable to that of liquid hydrogen. The hydrogenation and dehydrogenation of LOHCs are typically endothermic and exothermic, respectively. The dehydrogenation of dodecahydro-N-ethylcarbazole produces 22.1 wt % of hydrogen, which is a significant amount of heat, nearly 60% of the gravimetric energy density of diesel fuel. Therefore, hydrogenation and dehydrogenation of LOHC are more suitable for applications where heat can be conveniently utilized or supplied. Consequently, LOHCs are not suitable mobile applications. It is important to note that hydrogenation and dehydrogenation of LOHCs require a catalyst [172]. According to Jiang et al. [173], the top performing catalysts for the dehydrogenation of dodecahydro-N-ethylcarbazole contain, in ranking order, platinum, palladium, rhodium, gold, and ruthenium. The price of these precious metals is a major cost driver, and the monetary and environmental costs associated with the extraction of those metals are also significant [164,174]. Most LOHC systems involve catalysts based on noble metals. However, the use of catalysts based on non-noble-metals is possible in some cases. He et al. [175] studied the dehydrogenation of propane on a nickel-based catalyst. The activity of the catalyst decreased from 94% to 81% over a period of 82 h. The rapid activity decay is problematic for consumer or industrial applications, for which the catalysts are expected to last several thousands of hours before replacement is required.

It is interesting to note that these materials studied by Teichmann et al. are not acutely toxic [176], and are also insoluble in water, which is helpful in the event of a large-scale accidental spill. 

Hydrogen can also be stored as methylcyclohexane (MCH), which becomes toluene when dehydrogenated. NASA contemplated this approach in 1975 [177]. The “Spera” hydrogen is now commercialized [178], and has been used in a demonstration plant with capacity to convert 50 Nm^3^/h of hydrogen [179]. MCH contains 6 more hydrogen atoms than toluene. The molar mass of MCH is 98.19 g/mol, and it has the potential to release 6 wt % hydrogen or 5.5 MJ/L when converted to toluene. However, MCH is toxic [180] and lethal to rats. It may be ingested by marine life because of its water solubility [181]. Toluene is also toxic and can damage the nervous system [182]. 

Dibenzyltoluene is another LOHC which is being studied and evaluated [183,184,185,186,187,188]. It can store up to 6.2 wt % at 7.7 MJ/L of hydrogen [189], and it is a liquid substance at ambient temperature with low water solubility, flammability, and toxicity [190]. The reaction is strongly exothermic and must take place at high temperatures, which is not ideal for mobile applications. Again, a platinum catalyst is required for the conversions [191]. 

### 5.9. Chemical Hydrogen

It is evident that pure hydrogen is difficult to transport due to its physicochemical properties, such as low energy density. Therefore, it is worth investigating pathways to chemically store hydrogen. The term chemical hydrogen is used to describe the strategy of storing hydrogen by synthesizing molecules that contain hydrogen. Methane, the simplest hydrocarbon, can be synthesized by a process known as methanation. The global decarbonization trend spurred renewed interest in this process [192]. Methanation can be carried out biologically [193,194,195] or catalytically, from carbon monoxide or carbon dioxide. It produces hydrogen from electrolysis of water. After that, catalytic hydrogen–carbon dioxide methanation effectively converts electricity to methane [196,197,198]. While an industrial infrastructure for natural gas exists, the same cannot be said for methane. However, because natural gas is composed mostly of methane, the current natural gas technologies provide an acceptable estimation of the methane potential. The volumetric energy densities of hydrogen compressed at 700 bar and natural gas compressed at 250 bar are roughly the same. The volumetric energy density of liquefied natural gas is twice as high as that of hydrogen compressed at 700 bar. However, similar to liquid hydrogen tanks, liquid natural gas tanks are subject to boil-off, and methane is a potent greenhouse gas [199]. The chemical bonds between the carbon and hydrogen atoms are very stable, thus hydrogen is not readily extracted from methane. Therefore, methane must be utilized differently to pure hydrogen. A common way to produce hydrogen from methane is steam reforming, but this reaction is highly endothermic, i.e., it requires a lot of energy. Consequently, it is not suitable for mobile applications. Methane cracking is possible, but its energy balance is unclear [200]. Joglekar et al. [201] developed a direct methane fuel cell to produce electricity directly from methane. However, it uses a platinum catalyst and is far from commercial application. Solid oxide fuel cells (SOFCs) are perhaps the most interesting candidate for methane conversion [202]. One of the promising characteristics of SOFCs is their fuel flexibility [203]. Given the modest energy density of methane, other candidates should be considered for chemical hydrogen storage. 

**Figure 6:**
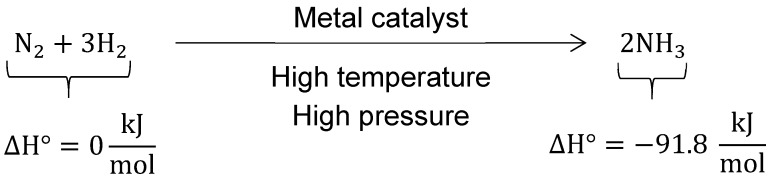
Haber–Bosch process summary [218].

Ammonia is another possible chemical hydrogen candidate that is being widely studied [204,205,206,207,208,209]. It can be produced without carbon dioxide [210] by the Haber–Bosch process (see Figure 6). The energy density of liquid ammonia (17.6 wt %, 11.5 MJ/L) is marginally better than that of liquid hydrogen, but its vapor pressure is much lower. The vapor pressure of ammonia is 10 bar at 25 °C [211], and this low pressure significantly simplifies the tank design. As evidence of that, there exists a widespread ammonia distribution and production infrastructure to process the millions of tons produced yearly around the globe. Similar to methane, ammonia utilization is more difficult than pure hydrogen. Solid oxide fuel cells are the most likely route for the use ammonia in fuel cells [212,213], but these fuel cells still encounter durability issues [214]. Some experts believe that SOFC will eventually establish itself as a dominating technology [215]. In the meantime, energy can be extracted from ammonia by combustion. There is a considerable amount of studies on the use of ammonia as a fuel [216]. Ammonia can be very harmful when inhaled in large quantities. However, it possesses a pungent smell, which may be considered a safety attribute. Additionally, it does not accumulate in the human body [217].

The production of methane, ammonia and other fuels from electricity is commonly referred to as “power-to-gas”. This group of technologies suffers from high cost and low efficiency [193]. 

## 6. Overview

Table 4 provides an overview of the storage methods. Because each storage methods possesses its own special characteristics, a direct comparison is difficult. Therefore, only typical figures are given to allow a rough comparison. 

## 7. Conclusions

Hydrogen is a practical energy vector for storage, and it can be used in conjunction with renewable energy use. For some mobility applications, it is possible to demonstrate that hydrogen has superior advantages compared to batteries, even if it is less energy efficient. Various hydrogen storage systems were presented, considering their application in the mobility industry. Compressed hydrogen cylinders are bulky and expensive, but they are the current choice of the industry for practical reasons, even if they do not achieve the DOE system target. Several drawbacks of the material-based hydrogen storage system were discussed. These include complex thermal management systems, expensive catalysts, stability issues, speed of kinetics, operating pressures, energy densities, and safety. Mostly due to the boil-off effect and poor efficiency, the suitability of liquid hydrogen for vehicles is questionable. As the leading industry standard, compressed hydrogen is far more developed than the other options. Its energy densities are 6.84 MJ/kg (5.7 wt %) and 4.90 MJ/L, whereas those of petroleum-based fuels are 45 MJ/kg and 35 MJ/L. Even if hydrogen is not as practical as petroleum, it represents a pathway to capture renewable energy and reduce fossil fuel consumption. 

## Figures and Tables

**Table 1:** Basic physico-chemical properties of hydrogen and natural gas.

Property	Hydrogen	Natural Gas
Lower heating value (LHV, MJ/kg)	120 [53]	52 [53]
Higher heating value (HHV, MJ/kg)	142 [53]	47 [53]
Density at 273 K (kg/m^3^)	0.09 [54]	0.65 [54]
Boiling point at atmospheric pressure(K)	20.3 [54]	111.2 [55]
Liquid density (kg/m^3^)	70.8 [54]	450.0 [55]
Flammability concentration limits in air (vol %)	4–75 [54]	5–15 [54]
Diffusion coefficient in air (cm^2^/s)	0.61 [54]	0.16 [54]

**Table 2:** Projected performance and cost of compressed automotive hydrogen storage systems compared to 2020 and ultimate DOE targets.

Storage System Targets	Gravimetric Density System (wt %)	Volumetric Density System (MJ/L)	Cost ($/kWh)
Current status (700 bar compressed)	4.2	2.9	15
2020	4.5	3.6	10
Ultimate	6.5	6.1	8

**Table 3:** Pressure vessel materials according to their type.

Type	Materials	Typical Pressure (bar)	Cost ($/kg)	Gravimetric Density (wt %)
I	All-metal construction	300	83	1.7
II	Mostly metal, composite overwrap in the hoop direction	200	86	2.1
III	Metal liner, full composite overwrap	700	700 [65]	4.2 [66]
IV	All-composite construction	700	633 [65]	5.7 (Toyota Mirai)

**Table 4:** Storage methods overview.

Method	Gravimetric Energy Density (wt %)	Volumetric Energy Density (MJ/L)	Temperature (K)	Pressure (barg)	Remarks
Compressed	5.7	4.9	293	700	Current industry standard
Liquid	7.5	6.4	20	0	Boil-off constitues major disadvantage
Cold/cryo compressed	5.4	4.0	40–80	300	Boil-off constitues major disadvantage
MOF	4.5	7.2	78	20–100	Attractive densities only at very low temperatures.
Carbon nanostructures	2.0	5.0	298	100	Volumetric density based on powder density of 2.1 g/mL and 2.0 wt % storage capacity.
Metal hydrides	7.6	13.2	260–425	20	Requires thermal management system.
Metal borohydrides	14.9–18.5	9.8–17.6	130	105	Low temperature, high pressure thermal management required
Kubas-type	10.5	23.6	293	120	
LOHC	8.5	7	293	0	Highly endo/exothermal requires processing plant and catalyst. Not suitable for mobility
Chemical	15.5	11.5	298	10	Requires SOFC fuel cell.
